# Outcomes of non-operative management of liver trauma in a low- and middle-income country: A single-centre experience from South Africa

**DOI:** 10.4102/jcmsa.v4i1.422

**Published:** 2026-07-20

**Authors:** Isabella M. Joubert, Mozamil Mohamed, Maeyane S. Moeng

**Affiliations:** 1 Department of Surgery, School of Clinical Medicine, Faculty of Health Sciences, University of the Witwatersrand, Johannesburg, South Africa

**Keywords:** liver trauma, non-operative management, polytrauma, lactate, AAST grade, LMIC, South Africa

## Abstract

**Background:**

Non-operative management (NOM) has become the standard of care for haemodynamically stable patients with liver trauma, yet robust data from low- and middle-income countries (LMICs) remain scarce, particularly in settings with a high burden of penetrating trauma.

**Methods:**

This single-centre retrospective cohort study analysed 251 adult patients with liver injuries managed non-operatively at a major trauma centre in Johannesburg, South Africa, between January 2017 and December 2023. The primary outcome was in-hospital mortality. Secondary outcomes included 30-day survival and associated injury patterns.

**Results:**

Overall mortality was 1.20% (3/251; Wilson 95% confidence interval [CI]: 0.41–3.45). All deaths occurred within 30 days. Admission lactate was the only variable significantly associated with mortality in the univariate analysis (odds ratio [OR]: 1.53 per 1 mmol/L increase, 95% CI: 1.11–2.10, *p* = 0.009). Sensitivity analysis using Firth’s penalised logistic regression yielded consistent results (penalised OR: 1.48, 95% CI: 1.08–2.03, *p* = 0.015). The American Association for the Surgery of Trauma (AAST) liver injury grade showed no association with mortality. Chest injuries were the most common associated injury (42.6%). Associated injury patterns were consistent across Grades III–V.

**Conclusion:**

In appropriately selected haemodynamically stable patients, NOM achieved excellent outcomes despite a substantial polytrauma burden. Physiology, rather than anatomic grade or associated injury pattern, determined survival. These findings support physiology-led selection in resource-constrained environments.

**Contribution:**

This study provides key insights into the real-world outcomes of non-operative management (NOM) of liver trauma in a high-volume, resource-constrained South African trauma centre. It demonstrates exceptionally low mortality (1.20%) with physiology-led patient selection despite a high burden of penetrating trauma and polytrauma. The primary contribution is evidence that admission lactate is a strong predictor of mortality, while anatomic injury grade (AAST) is not. These findings reinforce physiology-guided decision-making in LMIC settings and align directly with the journal’s focus on clinical practice and surgical outcomes in Southern Africa.

## Introduction

Trauma is a major global public health challenge and a leading cause of death and disability, accounting for approximately 4.4 million deaths annually, with more than 90% occurring in low- and middle-income countries (LMICs).^1,2^ In sub-Saharan Africa, including South Africa, the epidemiology of trauma is dominated by interpersonal violence and road-traffic collisions that disproportionately affect young males.^3,4^ These mechanisms frequently result in penetrating and blunt abdominal injuries. The liver, owing to its large size, anterior position and rich vascular supply, is the most injured solid abdominal organ.^5,6^

Historically, operative intervention was considered mandatory for nearly all liver injuries.^7^ However, over the past four decades, the management paradigm has shifted dramatically. Advances in multidetector computed tomography (CT) for accurate injury grading, the development of interventional radiology techniques such as angioembolisation and improvements in damage-control resuscitation and critical care have established non-operative management (NOM) as the preferred initial strategy for haemodynamically stable patients with both blunt and carefully selected penetrating liver injuries.^8,9,10^ In high-income countries (HICs), NOM success rates now routinely exceed 85–95%, with in-hospital mortality typically below 5%, even in high-grade (AAST IV–V) injuries when strict physiologic selection criteria are applied.^11,12,13^ Contemporary international guidelines from the Eastern Association for the Surgery of Trauma (EAST) and the World Society of Emergency Surgery (WSES) strongly recommend NOM as the standard of care in physiologically stable patients, regardless of anatomic grade.^14,15^

Nevertheless, outcomes and the applicability of NOM may differ substantially in LMIC settings such as South Africa. Penetrating trauma from interpersonal violence accounts for over 50–73% of liver injuries in urban trauma centres, pre-hospital transport times are often prolonged and access to advanced interventional radiology, intensive-care unit beds and blood products can be limited by resource constraints.^16,17,18^ Earlier South African series have shown that NOM is feasible, but they have also highlighted higher overall injury acuity and more variable utilisation rates compared with HIC cohorts.^19,20^ Although recent local data suggest that selected penetrating liver injuries can be managed non-operatively with low failure rates, detailed information on physiologic predictors of outcome, the influence of the American Association for the Surgery of Trauma (AAST) liver injury grade on survival, associated injury patterns and 30-day outcomes in contemporary LMIC practice remains limited.^21,22^

This knowledge gap is clinically significant. In resource-constrained environments, over-reliance on operative intervention can strain already limited operating theatre capacity and increase rates of non-therapeutic laparotomy, while inappropriate selection for NOM risks preventable mortality from delayed haemorrhage or missed associated injuries.^23^ This study addresses this need by providing a comprehensive description of the contemporary experience with NOM for liver trauma at a major South African trauma centre.

### Objectives

The primary objective of this study was to determine the in-hospital and 30-day mortality rates among patients with liver trauma who were managed non-operatively. The secondary objectives were:

- To identify clinical and laboratory predictors of mortality using descriptive comparisons and event-limited univariate analysis.
- To evaluate 30-day survival across AAST liver injury grades using the descriptive Kaplan–Meier survival analysis.
- To characterise baseline demographics, injury mechanisms, associated injury patterns and complications in this LMIC cohort, with specific attention to the consistency of polytrauma profiles across different liver injury grades.

## Research methods and design

Study design and setting: This was a retrospective cohort study conducted at a single high-volume tertiary trauma centre in Johannesburg, South Africa. The study period encompassed all eligible patients managed between January 2017 and December 2023.

Inclusion and exclusion criteria: All adult patients (> 18 years) with a documented liver injury who were managed non-operatively during the index admission were eligible for inclusion. Non-operative management was strictly defined as the absence of any laparotomy or operative intervention directed at the liver. Angioembolisation was performed in 12 patients (4.8%) without laparotomy; these patients were included in the NOM cohort. Patients who required immediate or early laparotomy because of haemodynamic instability, peritonitis or other surgical indications were excluded. Non-liver complications included complications not directly attributable to the hepatic injury itself, such as pulmonary, neurologic, infectious or systemic complications.

### Data collection

Data were extracted from the prospectively maintained institutional trauma registry using standardised fields. Variables collected included age, sex, mechanism of injury (blunt vs. penetrating), admission physiology (systolic blood pressure, mean arterial pressure, base excess, lactate), injury severity scores (Injury Severity Score [ISS] and New Injury Severity Score [NISS]), AAST liver injury grade according to the 2018 Organ Injury Scale, associated injuries and in-hospital complications.

### Outcomes

The primary outcome was in-hospital mortality. The secondary time-to-event outcome was 30-day survival, with censoring at hospital discharge or 30 days post-injury, whichever occurred first.

### Statistical analysis

Given the very low event rate (only three deaths), multivariable logistic or Cox regression models were considered statistically unreliable. Firth’s penalised logistic regression was performed as a sensitivity analysis for the primary predictor (admission lactate). Time-to-event data were analysed using the Kaplan–Meier survival curves stratified by AAST liver injury grade (III–V). All statistical tests were two sided, with *α* = 0.05.

### Ethical considerations

This study was conducted in accordance with the principles of the Declaration of Helsinki. Ethical clearance to conduct this study was obtained from the University of the Witwatersrand Human Research Ethics Committee (Medical) (No. M210236).

## Results

A total of 251 adult patients with liver injuries were managed non-operatively during the study period. Angioembolisation without laparotomy was performed in 12 patients (4.8%). The overall in-hospital mortality rate was 1.20% (3/251; Wilson 95% confidence interval [CI]: 0.41–3.45). All three deaths occurred within 30 days.

Non-survivors presented with significantly worse metabolic derangement on admission, evidenced by a median lactate of 9.9 mmol/L (interquartile range [IQR]: 6.7–10.2) compared with 2.7 mmol/L (IQR: 1.8–4.5) in survivors (*p* = 0.029). Non-liver complications were also significantly more frequent among non-survivors (66.7% vs. 10.1%, *p* = 0.031). There were no statistically significant differences between survivors and non-survivors in age, sex, mechanism of injury, systolic blood pressure, mean arterial pressure, base excess, ISS, NISS or AAST liver injury grade (Table 1).

**TABLE 1:** Baseline characteristics, injury profile and complications (overall and by outcome).

Variable	Overall (*n* = 251)				Survivors (*n* = 248)				Deaths (*n* = 3)				*p*-value
	Median	IQR	*n*	%	Median	IQR	*n*	%	Median	IQR	*n*	%	
Age (years)	32.0	26.5–37.0	-	-	32.0	26.8–37.0	-	-	35.0	29.5–39.0	-	-	0.731
Systolic BP (mmHg)	112.0	98.0–128.0	-	-	111.5	98.0–128.2	-	-	117.0	85.0–122.5	-	-	0.743
MAP (mmHg)	85.0	72.0–97.0	-	-	85.0	72.0–97.0	-	-	83.0	62.0–84.5	-	-	0.315
Base excess (mmol/L)	−4.3	−6.9 to -2.2	-	-	−4.2	−6.8 to −2.3	-	-	−10.0	−10.2 to -6.0	-	-	0.284
Lactate (mmol/L)	2.7	1.9–4.5	-	-	2.7	1.8–4.5	-	-	9.9	6.7–10.2	-	-	0.029
ISS	14.0	9.0–22.0	-	-	14.0	9.0–22.0	-	-	17.0	15.0–25.5	-	-	0.378
NISS	17.0	10.5–24.0	-	-	17.0	10.0–24.0	-	-	17.0	15.0–29.0	-	-	0.526
Liver AAST grade	3.0	2.0–3.0	-	-	3.0	2.0–3.0	-	-	3.0	2.5–3.0	-	-	0.777
Sex: Male	-	-	222	88.4	-	-	219	88.3	-	-	3	100.0	1.000
Injury type: Penetrating	-	-	141	56.2	-	-	140	56.5	-	-	1	33.3	0.583
Liver grade group: III	-	-	99	39.4	-	-	97	39.1	-	-	2	66.7	0.576
Liver grade group: IV–V	-	-	34	13.5	-	-	34	13.7	-	-	0	0.0	-
Any non-liver complication: Yes	-	-	27	10.8	-	-	25	10.1	-	-	2	66.7	0.031

ISS, Injury Severity Score; NISS, New Injury Severity Score; AAST, The American Association for the Surgery of Trauma; BP, blood pressure; MAP, mean arterial pressure.

Of the three non-survivors, two had major chest injuries (one haemopneumothorax requiring intercostal drainage and one pulmonary contusion with rib fractures) in addition to Grade III liver lacerations. The third patient had a severe traumatic brain injury (TBI) with a concomitant Grade III liver injury. In all three cases, death was attributed primarily to the extra-hepatic injuries and profound shock rather than exsanguination from the liver injury itself. No patient in the NOM cohort died of isolated liver-related haemorrhage.

**FIGURE 1:**
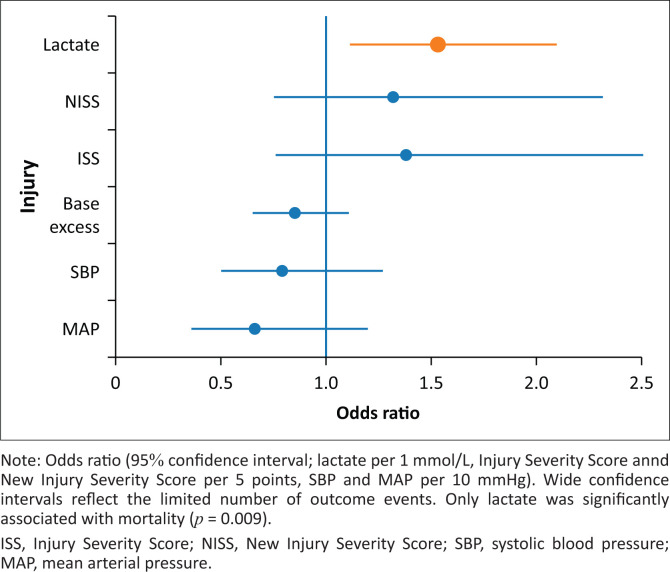
Univariate predictors of mortality in non-operative liver trauma.

Event-limited univariate logistic regression confirmed that admission lactate was the only variable significantly associated with mortality (odds ratio [OR]: 1.53 per 1 mmol/L increase, 95% CI: 1.11–2.10, *p* = 0.009). This association remained consistent on sensitivity analysis using Firth’s penalised logistic regression (penalised OR: 1.48, 95% CI: 1.08–2.03, *p* = 0.015). Injury severity scores and haemodynamic parameters showed only weak, non-significant associations (Table 2 and Figure 1).

**TABLE 2:** Univariate associations with mortality (event-limited model).

Predictor	OR	95% CI	*p*-value
Lactate (per 1 mmol/L)	1.53	1.11–2.10	0.009
NISS (per 5 points)	1.32	0.75–2.32	0.328
ISS (per 5 points)	1.38	0.76–2.51	0.291
Base excess (per 1 mmol/L)	0.85	0.65–1.11	0.221
SBP (per 10 mmHg)	0.79	0.50–1.27	0.334
MAP (per 10 mmHg)	0.66	0.36–1.20	0.177

OR, odds ratio; CI, confidence interval; ISS, Injury Severity Score; NISS, New Injury Severity Score; SBP, systolic blood pressure; MAP, mean arterial pressure.

**FIGURE 2:**
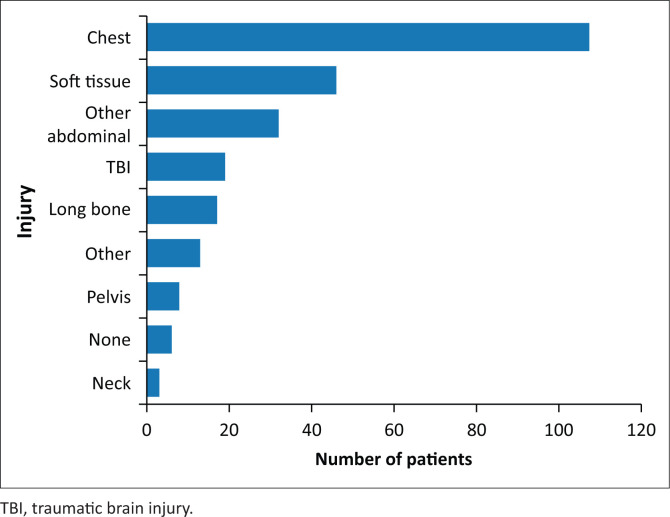
Distribution of associated injuries in non-operative liver trauma (non-operative management cohort).

Polytrauma was highly prevalent. The distribution of associated injuries is shown in Figure 2. Chest injury was the most common (*n* = 107, 42.6%), followed by soft tissue injury (18.3%), other abdominal injury (12.7%), TBI (7.6%), long bone injury (6.8%), other injuries (5.2%), pelvic injury (3.2%), no associated injury (2.4%) and neck injury (1.2%).

**FIGURE 3:**
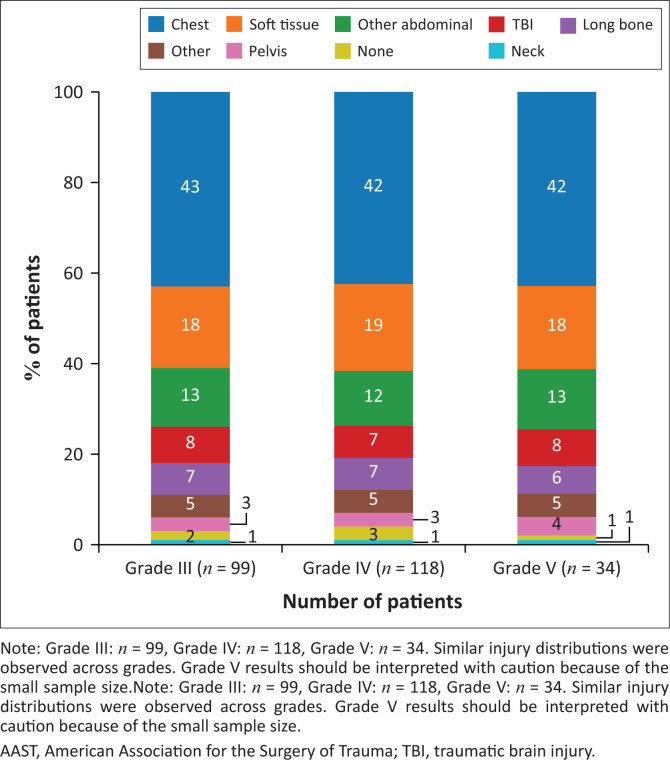
Stacked percentage distribution of associated injuries in non-operative liver trauma patients stratified by AAST liver injury grade (III–V).

Associated injury profiles were remarkably consistent across AAST liver injury Grades III–V. Chest injuries predominated in all grades. The percentage-normalised stacked distribution showed no meaningful shift with increasing liver grade (Figure 3).

**FIGURE 4:**
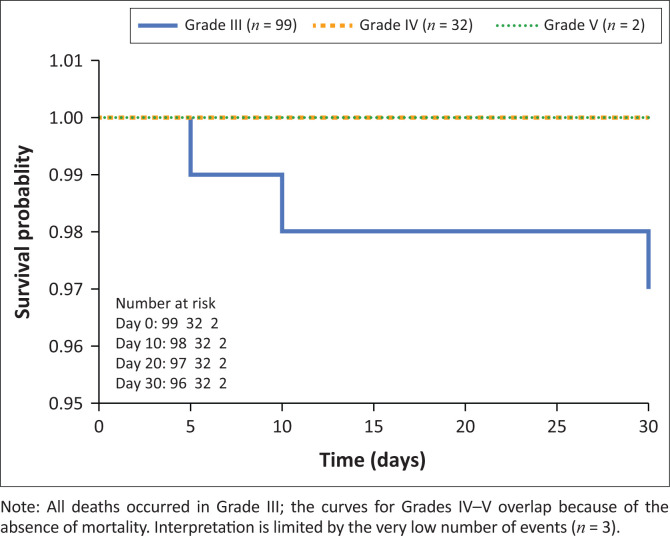
The Kaplan–Meier 30-day survival in non-operative liver trauma stratified by the American Association for the Surgery of Trauma (AAST) grade.

The Kaplan–Meier 30-day survival curves showed similarly high survival across Grades III–V although interpretation is limited by the very low number of events (Figure 4).

## Discussion

This large single-centre retrospective study from a high-volume trauma centre in Johannesburg, South Africa, demonstrates that NOM of liver trauma is both feasible and highly successful in a LMIC setting characterised by a high burden of penetrating trauma. With an in-hospital mortality rate of only 1.20% (3/251) among 251 patients selected for NOM, the outcomes achieved in this cohort are comparable to, or better than, those reported in contemporary HIC series.^11,12,13,15^

The predominance of penetrating injuries (56.2%) in this cohort reflects the local epidemiology of urban trauma in South Africa, where interpersonal violence remains a major driver of injury.^3,4^ This contrasts sharply with the blunt trauma predominance seen in most Western series. Despite this more challenging injury profile, prolonged pre-hospital transport times and resource constraints typical of LMIC settings, carefully selected patients achieved excellent results with NOM. These findings add important context-specific evidence to the existing literature and support the broader applicability of physiology-guided NOM even in environments with limited access to advanced interventional radiology and intensive care.^8,19,21^

A key finding of this study is that admission lactate was the only variable significantly associated with mortality on univariate analysis (OR: 1.53 per 1 mmol/L increase). This remained consistent on sensitivity analysis using Firth’s penalised logistic regression. Elevated admission lactate is a well-established marker of tissue hypoperfusion and systemic shock.^24^ In this series, non-survivors presented with profoundly elevated lactate levels (median 9.9 mmol/L), and all deaths were attributed primarily to extra-hepatic injuries (particularly severe chest trauma and TBI) and profound physiologic derangement rather than failure of liver haemorrhage control. This reinforces the critical importance of early metabolic monitoring and physiology-led decision-making in trauma care.^25^

Another important observation is the high prevalence of polytrauma, with chest injuries present in 42.6% of patients. Despite this substantial associated injury burden, overall mortality remained exceptionally low. This likely reflects careful physiologic selection for NOM and the inclusion of haemodynamically stable patients. Importantly, the patterns of associated injuries were remarkably consistent across AAST liver injury Grades III–V, indicating that differences in outcome were driven primarily by the patient’s physiologic response rather than the anatomic severity of the liver injury itself. The absence of mortality in the 34 patients with Grade IV–V injuries who were selected for NOM further challenges traditional anatomy-based decision-making and supports current international guidelines that recommend NOM for physiologically stable patients irrespective of injury grade.^14,15^

**FIGURE 5:**
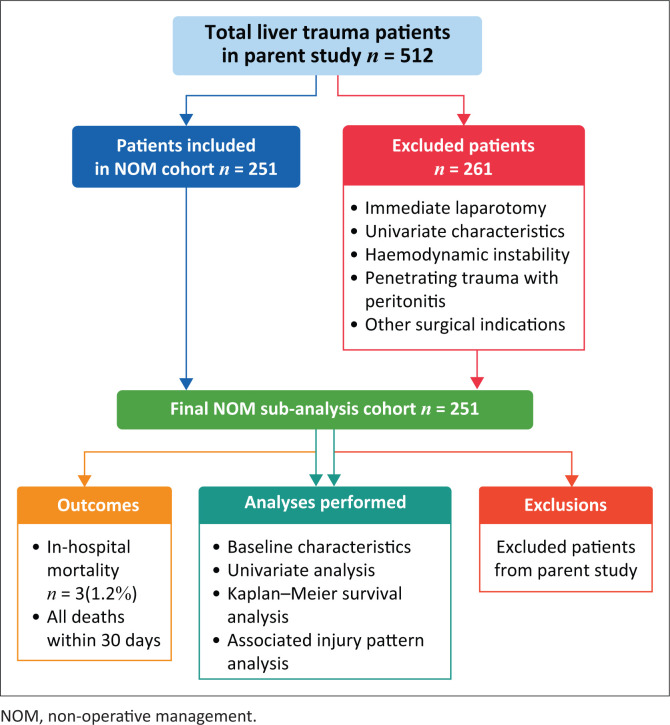
Flow diagram of the non-operative management sub-analysis.

The excellent outcomes observed in this study reflect careful physiologic selection for NOM rather than the universal applicability of this strategy to all patients presenting with liver trauma. Results apply only to haemodynamically stable patients who met institutional criteria for non-operative care included in the NOM cohort (see Figure 5). In resource-constrained environments, such careful selection is essential to avoid preventable mortality while optimising limited theatre and intensive care unit resources.

### Limitations

The retrospective, single-centre design and extremely low event rate (only three deaths) constitute the main limitations of this study. This severely restricts statistical power, precludes meaningful multivariable modelling and makes the results hypothesis-generating rather than definitive. Even univariate analyses should be interpreted with caution. Sensitivity analysis using Firth’s penalised likelihood regression produced consistent findings for lactate, but the overall fragility of results remains high. Selection bias inherent to NOM cohorts further limits generalisability. Prospective multicentre studies in similar LMIC settings are urgently needed.

## Conclusion

Non-operative management of liver trauma in appropriately selected haemodynamically stable patients at this South African trauma centre was associated with exceptionally low in-hospital mortality (1.20%) and near-perfect 30-day survival across all AAST injury grades. Admission lactate was significantly associated with mortality and may serve as a useful marker of physiological derangement. These results support the continued expansion and refinement of physiology-led NOM protocols in LMIC trauma systems, with emphasis on early identification of metabolic derangement, intensified monitoring and prompt escalation when indicated.

## References

[1] World Health Organization. Injuries and violence: The facts. Geneva: World Health Organization; 2024.

[2] Dixon J, Smith M, Jenkins L, et al. Preventable trauma deaths in the Western Cape of South Africa. PLoS Glob Public Health. 2024;4(5):e0003122. 10.1371/journal.pgph.0003122 38728269 PMC11086906

[3] Hardcastle TC, Oosthuizen G, Clarke DL, Lutge E. Trauma care in South Africa: An overview. S Afr Med J. 2015;105(9):789–792.

[4] Coccolini F, Coimbra R, Ordoñez C, et al. Liver trauma: WSES 2020 guidelines. World J Emerg Surg. 2020;15:24. 10.1186/s13017-020-00302-7 32228707 PMC7106618

[5] Kozar RA, Crandall M, Shanmuganathan K, et al. Organ injury scaling 2018 update: Spleen, liver, and kidney. J Trauma Acute Care Surg. 2018;85(6):1119–1122. 10.1097/TA.0000000000002058 30462622

[6] Moore EE, Cogbill TH, Jurkovich GJ, Shackford SR, Malangoni MA. Organ injury scaling: Spleen and liver (1994 revision). J Trauma. 1995;38(3):323–327. 10.1097/00005373-199503000-00001 7897707

[7] Stassen NA, Bhullar I, Cheng JD, et al. Nonoperative management of blunt hepatic injury: An Eastern Association for the Surgery of Trauma practice management guideline. J Trauma Acute Care Surg. 2012;73(5 Suppl 4):S288–S293. 10.1097/TA.0b013e318270160d 23114483

[8] Hommes M, Nicol AJ, Van der Vlies CH, et al. Management of blunt liver trauma in 134 severely injured patients in a high-volume trauma centre in South Africa. Injury. 2015;46(5):837–842. 10.1016/j.injury.2014.11.019 25496854

[9] Brooks A, Davies B, Smethhurst M, et al. Evolution of non-operative management of liver trauma. Trauma Surg Acute Care Open. 2020;5(1):e000551. 10.1136/tsaco-2020-000551 33178894 PMC7640583

[10] Van der Wilden GM, Velmahos GC, Emhoff TA, et al. Successful nonoperative management of the most severe blunt liver injuries. Arch Surg. 2012;147(5):423–428. 10.1001/archsurg.2012.147 22785635

[11] Saqib Y, Talving P, Branco BC, et al. Safety and efficacy of non-operative management in high-grade liver trauma: A systematic review. Eur J Trauma Emerg Surg. 2020;46(5):1055–1064.30719528

[12] Virdis F, Reccia I, Di Saverio S, et al. Clinical outcomes of non-operative management and observation in non-angioembolised hepatic trauma: A systematic review. Chin J Traumatol. 2022;25(4):201–208. 10.1016/j.cjtee.2022.04.004 35487854 PMC9458985

[13] Saltzherr TP, Van der Vlies CH, Van Lienden KP, et al. Improved outcomes in the non-operative management of liver injuries. Br J Surg. 2011;98(6):839–846. 10.1111/j.1477-2574.2011.00293.x PMC3093647 21492335

[14] Wilson EB. Probable inference, the law of succession, and statistical inference. J Am Stat Assoc. 1927;22(158):209–212. 10.1080/01621459.1927.10502953

[15] Peitzman AB, Heil B, Rivera L, et al. Blunt splenic injury in adults: Multi-institutional study of the Eastern Association for the Surgery of Trauma. J Trauma. 2000;49(2):177–185. 10.1097/00005373-200008000-00002 10963527

[16] Laing GL, Bruce JL, Skinner DL, et al. The burden of trauma at a district hospital in South Africa. S Afr Med J. 2014;104(9):611–615.

[17] Muckart DJ, Bhagwanjee S. American College of Surgeons trauma quality improvement programme versus South African trauma systems. S Afr Med J. 2016;106(10):1010–1012.27725022

[18] Nicol AJ, Hommes M, Primrose R, et al. Packing for control of hemorrhage in major liver trauma. World J Surg. 2007;31(3):569–574. 10.1007/s00268-006-0070-0 17334868

[19] Clarke DL, Thomson SR, Madiba TE, et al. Selective nonoperative management of liver trauma in a developing world environment. Br J Surg. 2005;92(7):906–910.

[20] Clarke DL, Allorto NL, Thomson SR. An audit of failed nonoperative management of abdominal trauma. Injury. 2010;41(5):488–491. 10.1016/j.injury.2009.10.022 19913226

[21] Navsaria PH, Nicol AJ, Edu S, et al. Selective nonoperative management of liver gunshot injuries. Ann Surg. 2009;249(4):653–656. 10.1097/SLA.0b013e31819ed98d 19300222

[22] Van Waes OJ, Cheriex KC, Navsaria PH, et al. Management of penetrating liver trauma in low-resource settings. World J Surg. 2012;36(4):807–813.22350477

[23] Leppäniemi A, Mentula P. Nonoperative management of abdominal trauma: A systematic review. Scand J Surg. 2015;104(3):146–152.25260783

[24] Vincent JL, Quintairos e Silva A, Couto L Jr, Taccone FS. The value of blood lactate kinetics in critically ill patients: A systematic review. Crit Care. 2016;20(1):257. 10.1186/s13054-016-1403-5 27520452 PMC4983759

[25] Dezman ZDW, Comer AC, Narayan M, Scalea TM, Hirshon JM, Smith GS. Lactate and other biomarkers as predictors of mortality in trauma patients: A systematic review. J Trauma Acute Care Surg. 2020;89(3):594–602.

